# Cost-effectiveness of camrelizumab plus rivoceranib for advanced hepatocellular carcinoma in the context of regional disparities in China

**DOI:** 10.3389/fonc.2024.1491404

**Published:** 2024-12-06

**Authors:** Zhonghua Zhao, Xiongying Jiang, Shiping Wen, Yanzhang Hao

**Affiliations:** ^1^ Department of Oncology, Binzhou Medical University Hospital, Binzhou, Shandong, China; ^2^ Department of Interventional Radiology, Sun Yat-sen Memorial Hospital, Sun Yat-sen University, Guangzhou, China; ^3^ Department of Minimally Invasive Interventional Therapy, State Key Laboratory of Oncology in South China, Guangdong Provincial Clinical Research Center for Cancer, Sun Yat-Sen University Cancer Center, Guangzhou, China

**Keywords:** cost-effectiveness, camrelizumab, rivoceranib, hepatocellular carcinoma, China

## Abstract

**Objective:**

To assess the cost-effectiveness of combining camrelizumab with rivoceranib versus sorafenib as initial treatment options for advanced hepatocellular carcinoma (HCC) across different developmental regions in China.

**Methods:**

Utilizing TreeAge Pro and data from the phase III randomized CARES-310 clinical trial, a model based on Markov state transitions was developed. Health state utility values were derived from the CARES-310 trial, and direct medical costs were obtained from relevant literature and local pricing data. The primary outcome measured was the incremental cost-effectiveness ratio (ICER), defined as the cost per additional quality-adjusted life year (QALY) gained per person. The ICERs were compared against the willingness-to-pay (WTP) thresholds of different regions in China, including low-income ($16,426.80), medium-income ($34,319.01), and high-income regions ($81,036.63). Sensitivity analyses were also conducted to assess the robustness and reliability of the model under various assumptions. A tornado diagram was used to illustrate the impact of parameter variations on the model’s cost-effectiveness.

**Results:**

For base case analysis, QALYs per person for the cohort receiving sorafenib were 0.91, with a corresponding cost of $8,860.97. For the cohort receiving camrelizumab plus rivoceranib, the QALYs per person were 1.71, with a corresponding cost of $16,190.72. The camrelizumab plus rivoceranib treatment group exhibited an increase of 0.80 QALYs and an additional expenditure of $7,329.75. The calculated ICER was $9,150.75 per QALY, which is below the WTP thresholds for all regions in China. The camrelizumab plus rivoceranib regimen is regarded as highly cost-effective in medium-income areas of China, with a probability of 99.9%. In high-income regions, the probability reaches 100.0%. Even in low-income regions, this regimen is considered 95.6% cost-effective. Sensitivity analysis further verified that these findings were robust across various assumptions.

**Conclusion:**

The combination of camrelizumab and rivoceranib as a treatment strategy not only improves health outcomes but also represents a cost-effective option across different developmental regions in China.

## 1 Introduction

Hepatocellular carcinoma (HCC) is the frequently seen digestive system cancer and the 4th leading cause of cancer-related death, with approximately half of the cases occurring in China (1). The rapid progression and high mortality rate of HCC make it a significant global public health issue. HCC ranks the fourth place among various cancers in China with regard to the prevalence, while the second in cancer-associated mortality (2). Because of the great malignancy grade and the diagnosis at the late stage, HCC has a low overall five-year survival rate, just 14.1% (3). Therefore, developing more effective treatment strategies, particularly for advanced HCC, is a primary focus of current medical research.

In recent years, immune checkpoint inhibitors (ICIs) have become the new treatment, which is safe and effective on treating HCC (4). Numerous clinical trials have demonstrated that ICIs, whether used alone or in combination with other treatments, are highly effective in enhancing tumor response rates and extending survival (5–8). These findings support that ICIs can be applied in treating advanced HCC.

Camrelizumab, a humanized IgG4 monoclonal antibody, is approved to be used to treat HCC in China (9). Its combination with the anti-angiogenic targeted drug rivoceranib has shown improved clinical efficacy and significant survival benefits (10). A Phase 3 CARES-310 randomized clinical trial further evaluated effectiveness between camrelizumab combined with rivoceranib and the conventional therapy sorafenib as the first-line treatment for advanced HCC patients (11). The results showed that camrelizumab plus rivoceranib remarkably enhanced median overall survival and progression-free survival relative to sorafenib, demonstrating superior survival advantages. However, the high costs associated with ICIs and the resulting economic burden have raised concerns about their cost-effectiveness (12). To allocate healthcare resources effectively and optimize HCC treatment strategies, it is essential to evaluate the economic benefits of new therapeutic approaches is essential.

This study assessed whether camrelizumab plus rivoceranib was more cost-effective than sorafenib as a first-line treatment in unresectable HCC patients in China, providing evidence for clinical decision-making. Considering the uneven regional development in China, this study further analyzes the results across different regions to provide more specific guidance for the rational treatment and drug selection for HCC, contributing to achieving more efficient allocation of healthcare resources.

## 2 Methods

This study did not involve patient enrollment, so informed consent was waived. Our study gained approval from ethics committee of this medical institution and was conducted following Consolidated Health Economic Evaluation Reporting Standards (CHEERS), **Supplementary Table 1** (12).

### 2.1 Patients and treatment

The study is based on the CARES-310 clinical trial that primarily focused on evaluating whether camrelizumab combined with rivoceranib was safer and more effective than sorafenib as an initial treatment to manage advanced HCC (11). Patient sample used for our study model matches the population evaluated in the clinical trial. Patients below were included: those developing Barcelona Clinic Liver Cancer stage B/C HCC, those not treated with surgery or local methods or showing progression after such treatments, and those not receiving previous systemic treatment.

Briefly, from 2019 to 2021, we included a total of 541 patients and randomized them into two treatment groups: camrelizumab plus rivoceranib (n=272) and sorafenib (n=269) groups. Patients received an intravenous injection of camrelizumab every two weeks at a dose of 200 mg, combined with either a daily oral dose of rivoceranib at 250 mg or a twice-daily oral dose of sorafenib at 400 mg. This treatment regimen continued in 28-day cycles unless terminated due to intolerable side effects, serious adverse events (AEs), or progressive disease. Most patients (88%) from camrelizumab plus rivoceranib group developed ≥ grade 3 AEs, relative to 182 patients (68%) in the sorafenib group. As in previous studies (13), the model just took grade 3/4 AEs occurring at the >10% frequency from each cohort. Based on disease progression, 90 patients (33%) in the camrelizumab plus rivoceranib group received subsequent systemic therapy, compared to 130 patients (48%) in the sorafenib group. Based on Phase 3 CARES-310 trial results, lenvatinib or sintilimab combined with bevacizumab was hypothesized to be the potential antitumor treatment in both groups, with best supportive care assumed for third-line treatment (11).

### 2.2 Model simulation

**Figure 1:**
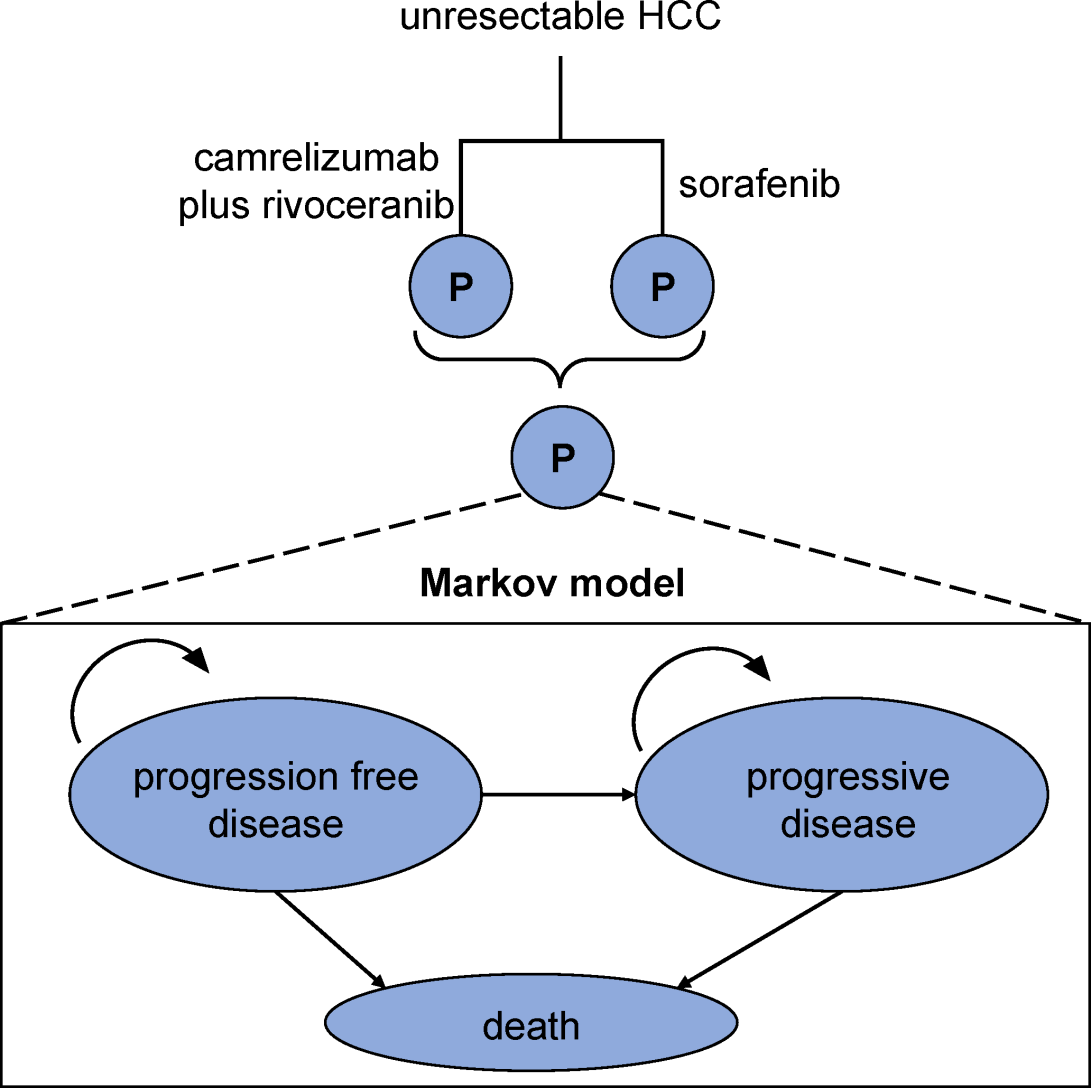
Model structure.

We used TreeAge Pro 2011 software to simulate outcomes in the 10-year follow-up duration. The semi-Markov model consists of three distinct and non-overlapping health states: non-progressive disease, progression-free disease, and mortality (**Figure 1**). Transitions between health states were derived according to CARES-310 trial findings (11). The simulation cycle in this study was set to 1 month, conforming to that of CARES-310 trial. Additionally, our analysis compared the willingness-to-pay (WTP) thresholds/quality-adjusted life year (QALY) per person across different regions: low-income ($16,426.80), medium-income ($34,319.01), and high-income regions ($81,036.63) (13).

### 2.3 State transition probabilities

**Figure 2:**
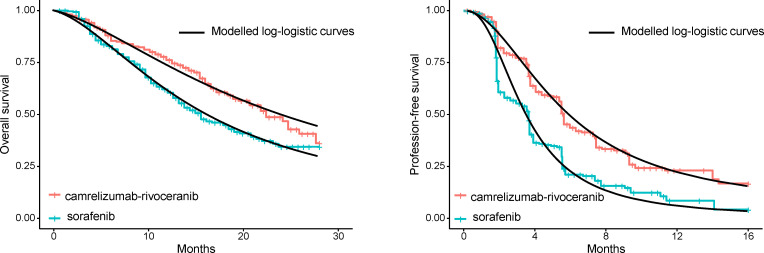
Survival curves in the trial and our modelled curves.

First, GetData Graph Digitizer was employed for extracting survival curves from CARES-310 clinical trial, obtaining survival probabilities and risk numbers at different time points for both groups. Subsequently, those curves obtained then underwent reconstruction with R software to obtain hypothetical individual patient data. Among the potential distributions, such as Weibull, log-normal, log-logistic, gamma, Gompertz, and exponential, we ultimately determined log-logistic distribution to offer best fit to simulate survival curves (**Supplementary Table 2** and **Figure 2**), thereby deriving health state transition probabilities at different time points. The survival function of a log-logistic distribution can be expressed as S(t) = 1/(1+λt^γ^).

### 2.4 Cost and utility inputs

**Table 1** displays more details of input parameters. This model incorporates various direct medical costs into the analysis, including drug costs, costs for managing severe AEs, follow-up care cost, follow-up therapy costs, and optimal supportive care costs. Drug cost data were obtained based on China Data Platform (https://data.yaozh.com/), whereas the rest cost data were obtained in related studies. To streamline the modeling process, this survival model just emphasized grade 3/4 AEs occurring at the >10% rate per treatment cycle of each treatment cohort. For evaluating roles of these variables in study outcomes, we conducted sensitivity analyses. The costs were transformed into U.S. dollars with the mean annual average rate for 2024 (1 USD = 7.05 RMB).

**Table 1:** Model inputs.

Parameters	Baseline value	Range	Distribution	Source
Survival inputs				
camrelizumab plus rivoceranib group OS survival model	**γ=**1.516, **λ=**0.008			(11)
sorafenib group OS survival model	**γ=**1.675, **λ=**0.010			(11)
camrelizumab plus rivoceranib group PFS survival model	**γ=**1.916, **λ=**0.036			(11)
sorafenib group PFS survival model	**γ=**2.277, **λ=**0.063			(11)
Adverse event incidence				
camrelizumab plus rivoceranib group hypertension	0.38	0.30 - 0.46	Gamma (90.25,0.0042)	(11)
camrelizumab plus rivoceranib group increased aspartate aminotransferase	0.16	0.13-0.19	Gamma (113.7778,0.0014)	(11)
camrelizumab plus rivoceranib group increased alanine aminotransferase	0.14	0.11 - 0.17	Gamma (87.1111,0.0016)	(11)
camrelizumab plus rivoceranib group decreased platelet count	0.11	0.09 - 0.13	Gamma (121,0.0009)	(11)
camrelizumab plus rivoceranib group palmar-plantar erythrodysaesthesia syndrome	0.12	0.10 - 0.14	Gamma (144,0.0008)	(11)
sorafenib group hypertension	0.15	0.12 - 0.18	Gamma (100,0.0015)	(11)
sorafenib group increased aspartate aminotransferase	0.05	0.04 - 0.06	Gamma (100,0.0005)	(11)
sorafenib group increased alanine aminotransferase	0.03	0.02 - 0.04	Gamma (36,0.0008)	(11)
sorafenib group decreased platelet count	0.01	0.01 - 0.01	Gamma (100,0.0001)	(11)
sorafenib group palmar-plantar erythrodysaesthesia syndrome	0.15	0.12 - 0.18	Gamma (100,0.0015)	(11)
Costs ($)				
camrelizumab (200mg)	438.19	350.55 - 525.83	Gamma (99.9954,4.3821)	https://data.yaozh.com/, accessed May 2024
rivoceranib (250mg)	15.67	12.53 - 18.80	Gamma (99.9362,0.1568)	https://data.yaozh.com/, accessed May 2024
sorafenib (200mg)	13.36	10.69 - 16.03	Gamma (100.1499,0.1334)	https://data.yaozh.com/, accessed May 2024
hypertension	1.39	1.11 - 1.67	Gamma (98.5765,0.0141)	(23)
palmar-plantar erythrodysaesthesia syndrome	17.13	13.70 - 20.55	Gamma (100.0584,0.1712)	(20, 24, 25)
increased aspartate aminotransferase	181.85	145.48 - 218.22	Gamma (100,1.8185)	(20, 24, 25)
increased alanine aminotransferase	89.92	71.94 - 107.90	Gamma (100.0445,0.8988)	(20, 24, 25)
decreased platelet count	342.11	273.69 - 410.54	Gamma (99.9912,3.4214)	(26)
subsequent lenvatinib therapy	650.76	520.61 - 780.92	Gamma (99.9954,6.5079)	(20)
subsequent sintilimab plus bevacizumab therapy	455.56	364.45 - 546.67	Gamma (100.0044,4.5554)	(24)
follow-up	60.98	48.78 - 73.17	Gamma (100.0164,0.6097)	(23)
best supportive care	367.71	294.17 - 441.25	Gamma (100.0054,3.6769)	(13)
Utility				
progression-free disease	0.76	0.61 - 0.91	Beta (23.8843,7.5424)	(13)
progressive disease	0.68	0.54 - 0.82	Beta (29.5176,13.8906)	(13)
death	0	0	Fixed	(13)
disutility due to hypertension	0.016	0.013 - 0.02	Beta (82.2384,5057.6596)	(20, 24, 25)
disutility due to palmar-plantar erythrodysaesthesia syndrome	0.15	0.12 - 0.18	Beta (84.85,480.8167)	(20, 24, 25)
disutility due to decreased platelet count	0.14	0.11 - 0.17	Beta (74.7756,459.3356)	(26)
**Discount rate**	0.03	0 - 0.05	Beta (5.5572,179.6828)	(13, 27, 28)

In a Markov model, cost calculation involved assigning costs to each health state and calculating the cumulative costs over time as patients transitioned between states. For the final output, cumulative costs were summed over all cycles and across all health states. The total cost represented the expected economic burden of the disease or intervention over the model’s time horizon, providing a basis for cost-effectiveness analysis when compared with corresponding health outcomes (e.g., QALYs). In summary, cost calculation in a Markov model integrated health state cost, transition probabilities, discounting, and sensitivity analysis to provide a comprehensive estimate of the economic impact associated with different treatment pathways.

We assessed life quality related to every health condition at the 0-1 scale, in which 0 stands for mortality, whereas 1 indicates the best health state. Due to the lack of explicit utility value data in CARES-310 trial, we obtained utility values in previous studies. Additionally, our model considered the negative impact of AEs on utility (13). Disutility was assumed to be additive, meaning it was subtracted from the utility values assigned to a given health state.

### 2.5 Sensitivity analysis

In conducting one-way sensitivity analysis, we systemically adjusted input parameters by ±20% for assessing the role of parameter alterations in incremental cost-effectiveness ratio (ICER: divide incremental cost by incremental effectiveness). Additionally, discount rates were 0%-5%. Tornado diagrams were drawn to effectively display the above analysis results.

Additionally, a comprehensive assessment of ICER uncertainty was conducted through probabilistic sensitivity analysis. This assessment involved executing 10,000 Monte Carlo simulations to include a broader array of potential probabilistic scenarios. To accurately represent cost parameters, a gamma distribution was employed, while a beta distribution was used to model utility value factors. Scatter plots were drawn to visually depict the results of this analysis.

## 3 Results

### 3.1 Base case

Regarding survival, the sorafenib group had a lower QALY gain of only 0.91 QALY per person (**Table 2**). In contrast, camrelizumab combined with rivoceranib demonstrated a significant improvement, reaching 1.71 QALY per person. In terms of costs, the overall cost of camrelizumab combined with rivoceranib group was $16,190.72, while that of sorafenib group was lower at $8,860.97.

**Table 2:** Base case results.

Variables	Sorafenib group	Camrelizumab plus rivoceranib group
Total cost ($)	8860.97	16190.72
QALYs	0.91	1.71
ICER ($/QALY)	/	9150.75
Monte Carlo analysis showing cost-effectiveness		
medium income region	0.10%	99.90%
high income region	0.00%	100.00%
low income region	3.30%	96.70%

ICER, Incremental cost–effectiveness ratio; QALY, Quality-adjusted life year.

Therefore, the treatment option of camrelizumab combined with rivoceranib led to an obvious incremental gain (0.80 QALY per person) in comparison with sorafenib. This means that patients receiving the camrelizumab plus rivoceranib treatment experienced the significantly improved overall life quality compared to those receiving sorafenib. However, this increase in effectiveness comes with a higher cost, with an incremental cost of $7,329.75 for the camrelizumab plus rivoceranib group in comparison with sorafenib group. Therefore, ICER was $9,150.75/QALY gained per person.

Overall, the ICER obtained in this analysis is below China’s WTP thresholds/QALY, including low-income ($16,426.80), medium-income ($34,319.01), and high-income regions ($81,036.63). This suggests that using camrelizumab plus rivoceranib can be the cost-effective and feasible treatment option for advanced HCC patients.

### 3.2 One-way sensitivity results

**Figure 3:**
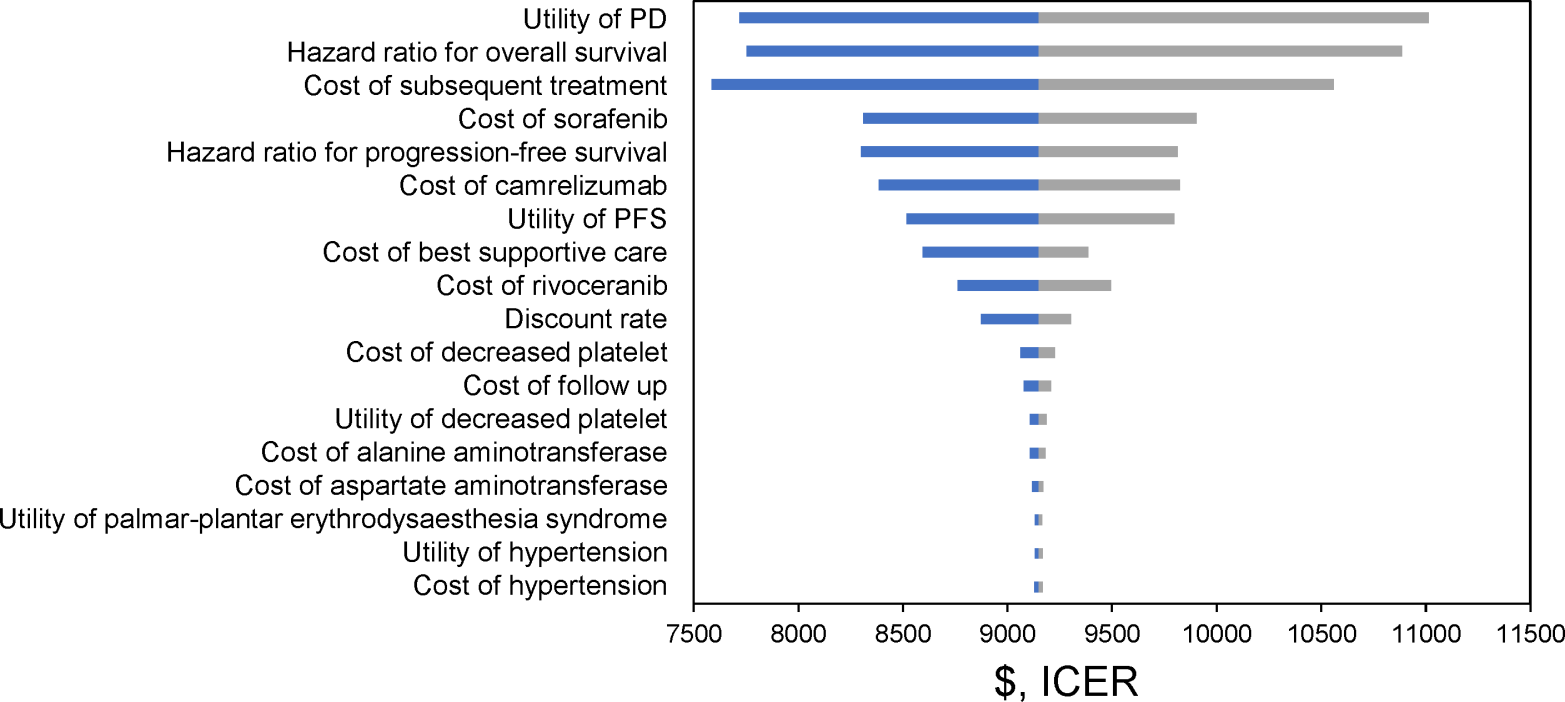
Tornado plot of one-way sensitivity analysis.

Tornado plot from **Figure 3** displays one-way sensitivity analysis results. Changing all input variables within a range of ±20% did not significantly affect our analysis results. The observation indicates that this model was robust and confirms that these findings do not qualitatively change even with fluctuations in these parameters.

### 3.3 Probabilistic sensitivity results

**Figure 4:**
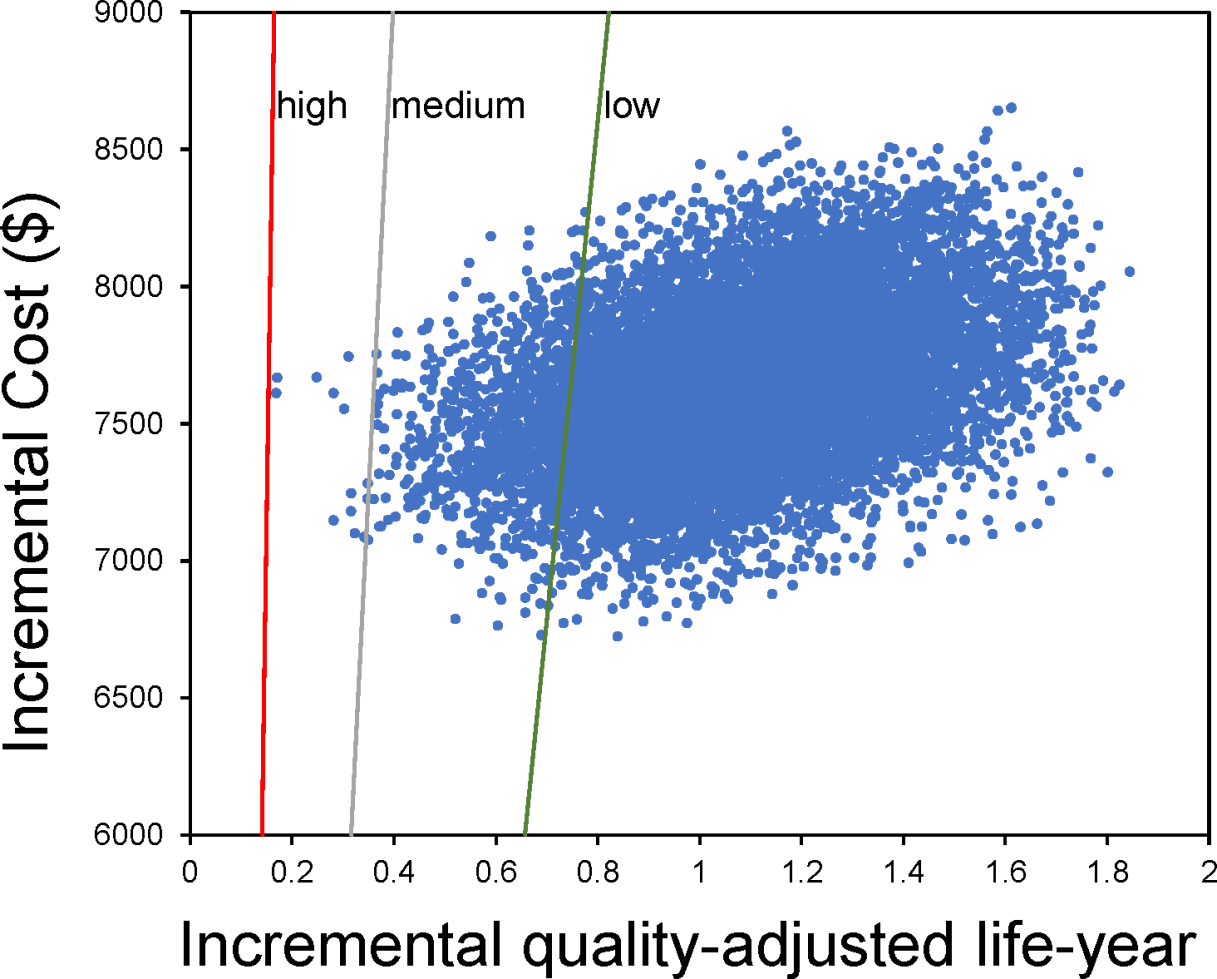
Scatter plot of the Monte Carlo simulations.

**Figure 4** presents the scatter plot results from Monte Carlo simulations. Upon the medium-income WTP threshold at $34,319.01/QALY, camrelizumab plus rivoceranib treatment would be considered the relatively cost-effective treatment, with the probability of 99.9% (**Table 2**). In high-income regions ($81,036.63), this probability reached 100.0%, and even in low-income regions ($16,426.80), the regimen was considered 95.6% cost-effective.

**Figure 5:**
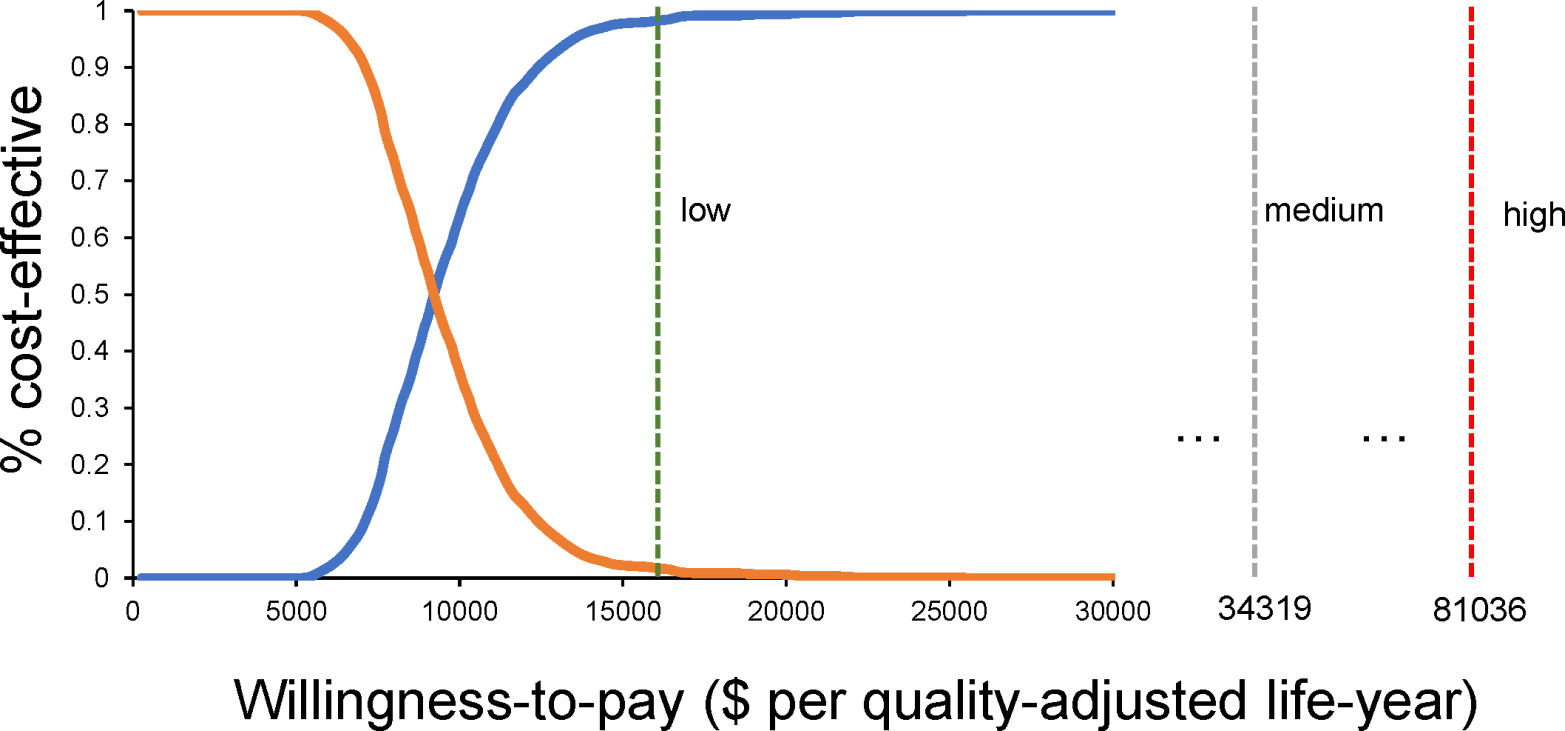
The probability that the camrelizumab plus rivoceranib regimen would be considered a more cost-effective regimen when WTP changes.

**Figure 5** shows that at a WTP of $9,150.75, camrelizumab plus rivoceranib and sorafenib are equally cost-effective, and with the increasing WTP value, the camrelizumab plus rivoceranib regimen demonstrates a health economic advantage.

## 4 Discussion

HCC accounts for the major factor leading to cancer-associated mortality globally, especially among those with advanced disease who face limited treatment options. In recent years, immunotherapy has demonstrated favorable outcomes in treating various cancers, including advanced HCC (14). In Chinese guidelines for the diagnosis and treatment of liver cancer (2024 edition), camrelizumab plus rivoceranib has been adopted to be the first-line therapy in advanced HCC patients. Given uneven regional development in China (15), determining whether camrelizumab combined with rivoceranib can be cost-effective among different regions of China is crucial. Our study is the first to find that the camrelizumab plus rivoceranib regimen is considered more cost-effective among medium-income areas in China, with a probability of 99.90%; in high-income regions ($81,036.63), it reaches 100.00%; even in low-income regions ($16,426.80), this regimen is considered 96.70% cost-effective. Therefore, this suggests that using the camrelizumab plus rivoceranib combination to be the first-line therapy option for advanced HCC may be cost-effective among different regions of China.

A three-state Markov model was utilized because, in cost-effectiveness analyses, increasing the number of states for advanced-stage patients can introduce potential drawbacks, specifically in the following areas: 1) limitations in the assumptions of transition probabilities, 2) challenges in parameter estimation, 3) difficulties in interpreting results, 4) risk of overlooking significant states, and 5) increased model complexity. It is crucial that the current model, with non-constant transition rates, is robust enough to accurately reflect the data. The log-logistic survival function, which aligns well with the observed trial duration, is assumed to implicitly capture survival patterns beyond the trial period.

There have been some economic studies regarding cost-effectiveness of additional ICIs for HCC, but subgroup analyses among different regions of China have not been conducted (16). Gong et al. comprehensively analyzed cost-effectiveness to evaluate efficacy among various regimens, including lenvatinib, sorafenib, atezolizumab plus bevacizumab, as well as sintilimab plus bevacizumab, in Chinese population (17). The study aimed to determine the QALYs generated by these treatments and assess their cost-effectiveness. The researchers found that lenvatinib, atezolizumab plus bevacizumab, and sintilimab plus bevacizumab therapies might be associated with significant incremental QALY gains compared to sorafenib. However, despite their clinical benefits, the above treatments were not considered cost-effective according to the WTP threshold of $36,600/QALY. Lang et al.’s study compared cost-effectiveness between camrelizumab combined with rivoceranib and sorafenib as the first-line treatment used to treat non-resectable HCC in China (18). Results showed that incremental benefit of camrelizumab combined with rivoceranib was 0.41 QALYs, while incremental cost was $13,684.84, leading to the ICER of $33,619.98/QALY, which is below China’s WTP threshold of $35,864.61/QALY, supporting that camrelizumab combined with rivoceranib was the cost-effective option. Wei et al. used CARES-310 trial-derived data for evaluating whether this combination therapy was cost-effective in comparison with sorafenib as a first-line treatment to treat unresectable HCC (19). ICERs for China and the United States and China were $30,410.56/QALY and $122,388.62/QALY, separately, both under their respective WTP thresholds ($35,898.87/QALY and $150,000/QALY separately), indicating that the combination therapy was cost-effective. Our study, based on different development regions, provides unique insights and complements existing evidence.

China is a country with uneven regional development, and promoting the availability and affordability of effective first-line treatment options can be beneficial for addressing healthcare inequalities (15). In many regions, especially among vulnerable populations, access to innovative treatments or specialized care is limited. By prioritizing cost-effective first-line therapies, healthcare providers can ensure that essential treatments are accessible to a larger population, regardless of socio-economic status or geographical location (20). The demand for effective and cost-effective first-line therapeutic options is becoming increasingly prominent in the treatment of HCC. With escalating healthcare costs and the need to optimize patient outcomes, it has become imperative to identify and utilize treatments that not only exhibit high efficacy but also offer affordability (21). The combination of camrelizumab and rivoceranib offers a dual-targeted approach, inhibiting both immune evasion and angiogenesis. The synergy between the immune-modulating effects of camrelizumab and the targeted inhibition of angiogenic pathways by rivoceranib may enhance treatment efficacy (7). Notably, the combination therapy has demonstrated a manageable safety profile, limiting treatment-associated toxicities. Our study shows that camrelizumab combined with rivoceranib was cost-effective as the viable initial treatment option for advanced HCC in China. The findings offer precious guidance to healthcare providers, policymakers, and other stakeholders involved in the management of advanced HCC, facilitating informed decision-making processes and optimizing the allocation of healthcare resources (22).

Currently, camrelizumab and rivoceranib have been approved for the treatment of HCC only in China, and studies regarding their use in other countries have not yet been published. Regarding safety, the most common grade 3 or 4 AEs reported in the CARES-310 trial included hypertension, palmar-plantar erythrodysesthesia syndrome, elevated aspartate aminotransferase (AST), and elevated alanine aminotransferase (ALT) (11). In the monotherapy and combination therapy groups, 66 cases (24%) and 16 cases (6%) of treatment-related serious AEs were reported, respectively. There was one treatment-related death in each group: multi-organ dysfunction syndrome in the combination therapy group and respiratory and circulatory failure in the monotherapy group. Approximately half of the patients experienced hypertension, but in most cases, it was manageable, and only a few patients discontinued treatment due to hypertension. Regarding hepatic toxicity, grade 3 or higher hepatic AEs (e.g., ascites or hepatic encephalopathy) were more common with the camrelizumab + rivoceranib combination than with sorafenib alone, possibly due to cumulative drug-induced liver damage. However, patients’ overall liver function remained stable throughout the combination treatment. Additionally, the incidence of grade 3 or 4 AEs in patients using sorafenib in this study was high, possibly due to the inclusion of patients more susceptible to adverse events, with an ECOG performance status of 1 and a higher baseline proportion of patients previously treated with locoregional therapies. This factor may also have contributed to the increased risk of liver toxicity observed in both the sorafenib and camrelizumab + rivoceranib treatment groups.

This study has the following limitations. First, after the follow-up period in CARES-310 trial, OS curves were immature for camrelizumab combined with rivoceranib and sorafenib (median OS had not been reached). Considering the role of survival curves in ICER, updating current model results is essential in the future if new data can be obtained. Second, due to rigid eligibility criteria and controlled treatment protocols, our pharmacoeconomic assessment may not be generalizable. Third, prices of camrelizumab and rivoceranib may vary because of changes of dosage forms, marketing protocols and indications. Lastly, subsequent treatment protocols were simplified based on clinical practice and CARES-310 trial. In real world, second-line treatments can be highly complicated, which significantly affect the experimental results. Despite these limitations, it is feasible to analyze the cost-effectiveness on the basis of CARES-310 trial data, which can offer precious data for making treatment decision.

## 5 Conclusions

Based on this study, camrelizumab plus rivoceranib shows higher cost-effectiveness compared with sorafenib in China, even among low-income regions. Our findings provide precious references for decision-makers regarding the use of camrelizumab and rivoceranib.

## Data Availability

The original contributions presented in the study are included in the article/**Supplementary Material**. Further inquiries can be directed to the corresponding authors.
